# Nutritional Interventions in the Management of Fibromyalgia Syndrome

**DOI:** 10.3390/nu12092525

**Published:** 2020-08-20

**Authors:** Giuditta Pagliai, Ilaria Giangrandi, Monica Dinu, Francesco Sofi, Barbara Colombini

**Affiliations:** 1 Department of Experimental and Clinical Medicine, University of Florence, 50134 Florence, Italy; giangrandii@aou-careggi.toscana.it (I.G.); monica.dinu@unifi.it (M.D.); francesco.sofi@unifi.it (F.S.); barbara.colombini@unifi.it (B.C.); 2 Unit of Clinical Nutrition, Careggi University Hospital, 50134 Florence, Italy

**Keywords:** fibromyalgia, diet, nutrition, food, review

## Abstract

Fibromyalgia (FM) is a multifactorial syndrome of unknown etiology, characterized by widespread chronic pain and various somatic and psychological manifestations. The management of FM requires a multidisciplinary approach combining both pharmacological and nonpharmacological strategies. Among nonpharmacological strategies, growing evidence suggests a potential beneficial role for nutrition. This review summarizes the possible relationship between FM and nutrition, exploring the available evidence on the effect of dietary supplements and dietary interventions in these patients. Analysis of the literature has shown that the role of dietary supplements remains controversial, although clinical trials with vitamin D, magnesium, iron and probiotics’ supplementation show promising results. With regard to dietary interventions, the administration of olive oil, the replacement diet with ancient grains, low-calorie diets, the low FODMAPs diet, the gluten-free diet, the monosodium glutamate and aspartame-free diet, vegetarian diets as well as the Mediterranean diet all appear to be effective in reducing the FM symptoms. These results may suggest that weight loss, together with the psychosomatic component of the disease, should be taken into account. Therefore, although dietary aspects appear to be a promising complementary approach to the treatment of FM, further research is needed to provide the most effective strategies for the management of FM.

## 1. Introduction

Fibromyalgia (FM) is a complex, multifactorial syndrome characterized by widespread chronic pain and a constellation of somatic and psychological manifestations, including fatigue, joint stiffness, sleep disorders, depression, anxiety, gastrointestinal and cognitive disorders [1,2,3]. Diagnostic criteria have recently been updated by the American College of Rheumatology and, using these criteria, FM is now recognized as one of the most common chronic pain conditions and the second most common cause of visits to rheumatologists after osteoarthritis [4]. It can occur at any age with a prevalence of 2–8% in the general adult population, and is more common in women than men, with a ratio of 2:1 [5,6]. Despite progress in understanding the mechanisms involved, the etiology of FM is still unknown, and the pathophysiology uncertain. Various evidence supports the hypothesis that FM is a “central pain disorder”, with alterations in central nervous system function leading to increased nociceptive processing [7,8]. Furthermore, recent evidence suggests that low-grade systemic inflammation, a preponderance of prooxidative status and an insufficient antioxidant capacity, could contribute to the development of the disease, reducing the pain threshold and inducing fatigue and mood disorders [9,10]. FM shows strong family aggregation and, although this may be due to shared environmental or behavioural influences, twin studies have revealed that genetic variants and hereditary mechanisms contribute to 50% of the risk of developing chronic pain and related conditions in FM [11,12,13]. Currently, no predisposing gene has been found, but it has been reported that several environmental factors, such as psychological and physical trauma or certain infections, can trigger development and influence the severity of FM through epigenetic mechanisms [14,15]. It is believed that there is a bidirectional temporal association between some psychological stressors and FM, with an increased risk of developing each other, suggesting a potential shared pathophysiological mechanism underlying these different conditions [16]. 

To date, optimal management of FM requires a timely diagnosis, together with a comprehensive assessment of pain, function and psychosocial context. Effective treatment is not available, and experts recommend nonpharmacological therapy as a first-line strategy, with the pharmacological option to be chosen only in case of lack of effect [17,18]. Among the nonpharmacological treatment options, nutrition has shown increasing interest in the literature in recent years [19,20,21]. This review aims to summarise the possible relationship between FM and nutrition, exploring the role of nutrients, foods and dietary patterns in FM syndrome.

## 2. Nutritional Supplementation and Fibromyalgia 

An imbalance of dietary components, including minerals and vitamins, can play a critical role in the development of FM. A survey conducted by Arranz and colleagues [22] revealed that 73% of the subjects affected by FM are users of nutritional supplements, and 61% of these became users after the onset of the disease. A recent meta-analysis showed little evidence to support the hypothesis that vitamin and mineral deficiencies could play a significant role in the development of FM, or that the use of nutritional supplements could be effective in these patients [23]. However, some intervention studies evaluating the possible effects of nutritional supplements on FM patients have been published to date and are reviewed in Table 1.

### 2.1. Vitamin D

Some evidence suggests that vitamin D supplementation should be considered in the management of FM in light of the fact that about 40% of FM subjects have been reported with vitamin D deficiency [37]. In addition, several studies have suggested an association between low serum vitamin D levels and chronic pain, depression and anxiety in FM patients [38,39]. The first study investigating the effect of vitamin D supplementation on FM subjects was conducted by Arnold and colleagues in 2008 [25]. Ninety FM patients with mild to moderate vitamin D deficiency were randomly assigned to receive 50,000 units of cholecalciferol (vitamin D_3_) per week compared with a placebo. After 8 weeks of intervention, the treated group showed a significant improvement in FM scores, in contrast to the placebo group [25]. Subsequently, other studies were performed to evaluate the effect of vitamin D supplementation in FM patients [26,27,28,29,30,31]. Although in a limited sample of participants, all of these studies reported a beneficial effect of vitamin D supplementation. Only the study by Warner et al. reported inconclusive results regarding the benefits on FM symptoms [24]. However, all authors stressed the importance of testing serum vitamin D levels and recommend supplementation when risk factors for vitamin D deficiency are present.

### 2.2. Vitamin C and Vitamin E

Antioxidant vitamins such as vitamin C and vitamin E can play a beneficial role in the management of certain typical symptoms of FM, since they are useful for preserving the cerebellar functions, memory, emotive responses as well as muscle function [40]. However, there are currently no consistent studies in the literature. A recent meta-analysis reported lower levels of vitamin E in FM patients than in healthy controls, although this difference disappeared when low quality studies were excluded from the analysis [23]. In addition, treatment with vitamin C and E combined with or without exercise compared to exercise only in 32 women with FM over a 12 week period did not show a statistically significant improvement in FM symptoms, although both interventions resulted in significantly higher serum levels of vitamin A, C and E [32].

### 2.3. Minerals

Regarding the mineral status, several studies have shown a decrease in intracellular magnesium content in FM patients [41,42]. Magnesium deficiencies were largely associated with low-grade inflammation, muscle weakness and paresthesia, which are typical symptoms of FM [21]. A recent study has shown that low dietary magnesium intake is correlated with worsening pain threshold parameters in FM patients [43]. Magnesium has always been considered the nonpharmacological supplement with the highest potential for FM [44]; however, so far, only two clinical trials have been performed in FM patients. The first study investigating the effect of magnesium combined with malic acid supplementation was performed in 1995 by Russell and colleagues, showing little or no effect on pain or depression in 24 women with FM when using low doses of supplementation [33]. However, with increased dose and a longer duration of treatment, a significant improvement in pain and tenderness was reported [33]. The second study tested the effect of treatment with magnesium citrate in combination with amitriptyline versus amitriptyline in 60 female FM subjects, showing that amitriptyline and magnesium supplementation were more effective in all measured outcomes than amitriptyline alone [34]. 

With regard to other minerals, some studies indicated a potential link between iron deficiency and FM [45,46,47]; however, only one study evaluated the effect of iron supplementation on FM symptoms, fatigue and iron status of 81 FM subjects, showing an overall improvement only in the treated group [35].

### 2.4. Probiotics

Increasing evidence suggests that FM patients may present altered microbiota, with the abundance of different taxa selectively correlated with disease-related symptoms [48]. This has led researchers to hypothesize a potential beneficial use of probiotics in the treatment of FM. A pilot study to investigate the effect of a 7 week supplementation with a multispecies probiotic showed improved cognition, particularly impulsive choice and decision-making, in 40 subjects diagnosed with FM [36]. On the other hand, no other beneficial effects were observed in self-reported pain, quality of life, depression or anxiety [36].

### 2.5. Other Substances

Several studies have reported an association between amino acid deficiencies such as valine, leucine, isoleucine and tryptophan and FM symptoms [49,50]. However, to date, no intervention studies have been carried out to test the effect of a supplementation with these elements. Furthermore, some studies have reported some potential benefits in FM patients from botanical or antioxidant supplements, although the evidence to support these substances is very weak. Several nutritional supplements such as Chlorella pyreinoidosa, cellfood, coenzyme Q10, Ginkgo biloba, ascorbigen, L-carnitine, S-adenosylmethionine, creatine and melatonin have shown some benefits for FM patients, improving symptoms such as muscle pain, fatigue, morning stiffness and quality of life [19]. Although patients frequently experience several positive effects from supplementations, there is insufficient evidence to recommend their use in the clinical practice.

## 3. Dietary Interventions and Fibromyalgia

Several dietary approaches have been proposed with the aim of reducing the symptomatology of FM (Table 2). The proposed nutritional strategies have been usually aimed to correct any nutritional deficiencies or to interfere with the different pathophysiological pathways supposed to be involved in the occurrence of FM.

### 3.1. Olive Oil

Extra-virgin olive oil (EVOO) is characterized by a high concentration of phenolic compounds. The countless health benefits of the EVOO are mainly due to its antioxidant activity, which is linked to its ability to protect DNA, proteins and lipids from damage caused by exposure to reactive oxygen species (ROS), which in turn are increased in FM patients [51]. A clinical trial investigated the effect of 50 mL/die of EVOO compared to refined olive oil in 23 female FM subjects. After 3 weeks of intervention, the authors reported a statistically significant improvement in protein carbonylation, lipid peroxidation, FIQ and mental health status after the intervention with EVOO [51]. Recently, the same research group reported similar beneficial effects of EVOO on several cardiovascular risk markers of 30 FM women, concluding that EVOO can protect women with FM against cardiovascular disease, thus proving to be a valuable therapeutic support in patients with FM [52].

### 3.2. Ancient Grain Products

In recent years, interest in ancient grains such as Khorasan wheat has been steadily increasing due to their beneficial effect on various pathological conditions [73,74]. The positive effect on health status seems to be due to the higher content of macro- and microelements, in particular magnesium, phosphorus, potassium, selenium and zinc, as well as carotenoids and polyphenols, compared to modern wheat [75]. Our group recently studied the effect of a replacement diet with cereal products based on ancient Khorasan wheat compared to the modern variety “Palesio” on the symptoms and quality of life of 20 patients with FM [53]. After 8 weeks, participants reported an overall improvement in the severity of FM-related symptoms, including widespread self-reported body pain, daytime sleepiness, fatigue and tiredness, resulting in an improvement in the impact of the disease on daily activities, with a greater positive effect after the intervention with Khorasan.

### 3.3. Monosodium Glutamate and Aspartame-Free Diet

Monosodium glutamate (MSG) and aspartame may act as excitotoxin molecules in organisms, acting as excitatory neurotransmitters, and may lead to neurotoxicity if used in excess [76]. Two case series on a total of 6 FM patients reported an overall improvement in FM symptoms such as chronic pain, fatigue, sleep and cognitive function after several months of aspartame-free or MSG plus aspartame-free diet [76,77]. A 30% remission of symptoms after an excitotoxin elimination diet was also observed in a sample of 46 patients with FM and irritable bowel syndrome (IBS) [54]. Interestingly, the MSG challenge led to a significant return of symptoms, a worsening of FM severity and a decrease in quality of life in almost all patients [54]. On the other hand, a total of 36 women with FM reported no significant differences in pain after a 12 week elimination of dietary MSG and aspartame, suggesting that discontinuation of dietary MSG and aspartame did not improve the symptoms of FM [55].

### 3.4. Gluten-Free Diet

FM patients often have gastrointestinal symptoms that significantly overlap with various gluten-related disorders such as nausea, abdominal pain, fatigue, tiredness, chronic pain and mood disturbance, suggesting a possible coexistence of noncoeliac gluten sensitivity in such patients [18]. This has led many investigators to hypothesize that a gluten-free diet could be beneficial for patients with FM. A pilot study investigating the clinical impact of a 1 year gluten-free diet in a small sample of 7 patients with coeliac disease, IBS and FM, revealed an overall improvement of pain symptoms, quality of life, cognitive function as well as of tissue transglutaminase serum levels [56]. The same research group investigated the effect of a 1 year gluten-free diet on 97 women with FM and IBS with or without lymphocytic enteritis, showing a slight but significant improvement in both IBS-related symptoms (chronic abdominal pain, changes in intestinal habit, bloating) and FM-related symptoms (chronic widespread pain, generalized tender points, fatigue and restless sleep) in the subgroup of lymphocytic enteritis [57]. Similar results were obtained in a 16.4 month gluten-free intervention on 20 FM patients without coeliac disease [58]. In addition, Slim and colleagues recently performed a 6 month intervention trial to study the effect of a gluten-free diet versus a low-calorie diet in 75 patients with FM experiencing gluten sensitivity-like symptoms [59]. Authors found that both dietary interventions resulted in beneficial effects on symptoms concluding that the gluten-free diet was not superior to the low-calorie diet in FM subjects with symptoms similar to gluten sensitivity [59].

### 3.5. Low-FODMAPs Diet

FODMAPs (Fermentable Oligo-Di-Mono-saccharides And Polyols) are poorly absorbed short-chain carbohydrates including lactose, free fructose, polyols, fructans and galacto-oligosaccharides. The low-FODMAPS diet has shown significant benefits in the treatment of IBS [78]. Since 70% of FM patients suffer from IBS [79], it has been hypothesized that the low-FODMAPs diet could be beneficial for FM subjects [60,61]. A 4 week intervention trial of 38 women with FM showed a significant reduction in gastrointestinal disorders and FM symptoms, including pain scores [60], as well as a reduction in body weight and waist circumference [61].

### 3.6. Low-Calorie Diet

It is known that high body mass index is associated with a number of disabling musculoskeletal conditions, suggesting that obesity may worsen symptoms of FM [62]. The most commonly used dietary strategy to reduce body weight is certainly the caloric restriction. In a pilot study, Shapiro and colleagues tested the effect of a low-calorie diet on 42 patients with FM, showing that after 20 weeks of intervention, participants reported a 4.4% reduction in body weight, along with an improvement in pain symptoms, body satisfaction and quality of life [62]. Similar results were obtained years later by Senna et al. [63], analyzing the effect of a 6 month hypocaloric diet on 83 FM subjects. Patients who lost weight reported lower interleukin-6 and C-reactive protein levels than controls, as well as lower depression and improved sleep and quality of life [63]. As a result, a more aggressive low-calorie diet for 12–16 weeks in 123 obese FM subjects led to improved pain symptoms, sleep pattern and depression, along with increased levels of anti-inflammatory cytokine interleukin-10 [64].

### 3.7. Vegetarian Diet

Vegetarian diets are characterized by large amounts of plant foods rich in fiber, vitamins, minerals and antioxidant elements, leading to the hypothesis that this dietary pattern may exert pain-relieving effects in FM patients, due to its anti-inflammatory properties and absence (or reduction) of animal proteins [80,81]. The first study testing the effect of a vegetarian diet on FM patients was performed in 1993 on a small sample of 10 FM patients [65]. After a 3 week period of vegetarian diet, participants reported an overall improvement in subjective well-being. Some years later, Kaartinen et al. tested a 3 month strict raw vegan diet on 18 FM patients, highlighting a significant improvement in pain scores, joint stiffness and sleep quality [67]. Interestingly, these beneficial effects tended to disappear immediately after shifting back to the omnivorous diet. Similar results with a raw vegan diet were obtained by Hänninen et al. on a group of 33 FM patients after a 3 month intervention period [68] and by Donaldson and colleagues on a sample of 30 FM subjects followed for 7 months [69]. On the other hand, after 6 weeks of dietary intervention with a vegetarian diet, 37 FM patients reported a significant improvement in pain symptoms, but this turned out to be smaller than in a control group of patients receiving a pharmacological treatment with amitriptyline [66]. Finally, a 4 week intervention program combining core stabilization exercises plus a lacto-vegetarian diet in 21 patients with FM who had low back pain led to pain reduction and improved body composition [70].

### 3.8. Mediterranean Diet

Only little evidence is available on the possible beneficial effects of the Mediterranean diet on FM. A recent cross-sectional study of 95 FM women showed that adherence to the Mediterranean diet was consistently associated with quantitative calcaneal ultrasound parameters, supporting the hypothesis that adherence to the Mediterranean diet may play a determining role in bone health in FM women [82]. Given that alterations in the intestinal bacterial flora appear to be a contributing factor in many chronic inflammatory and degenerative diseases, including rheumatic diseases such as FM, Michalsen and colleagues have tested the effect on the gut microbiota of interventions with the Mediterranean diet or a modified intermittent 8 day fasting regimen in 35 patients affected by FM [71]. Surprisingly, after 2 weeks and 3 months of follow-up, the authors found no significant changes in fecal bacteria counts following the two dietary interventions within and between groups. In addition, no significant differences appeared in the analysis of secretory immunoglobulin A or the symptomatology, suggesting that neither Mediterranean diet nor fasting treatments influenced the gut microbiota or symptoms in FM patients [71]. On the other hand, a recent study of 22 FM patients revealed that a 16 week Mediterranean diet with or without high doses of tryptophan and magnesium led to several beneficial effects on emotional processing, decreased fatigue, anxiety and depression, and reduced possible eating disorders and body image dissatisfaction, with significant greater improvements especially in the Mediterranean diet plus supplements group [72].

## 4. Conclusions

This review embraced the literature and showed that the role of dietary supplements on FM remains controversial, although clinical trials with vitamin D, magnesium, iron and probiotics’ supplementation show promising results. In terms of dietary interventions, the administration of olive oil, the replacement diet with ancient cereals, low-calorie diets, vegetarian diets, the low-FODMAPs diet, the gluten-free diet, the monosodium glutamate and aspartame-free diet and the Mediterranean diet all appear to be effective in reducing the symptoms of FM. The majority of the included studies showed a significant improvement in chronic pain, anxiety, depression, cognitive function, sleep pattern and gastrointestinal symptoms. In addition, weight loss seems to be associated with both reduced inflammation and improved quality of life in FM subjects, thus suggesting that body weight could have a functional repercussion in these patients. Therefore, the fact that the improvement has been achieved through different dietary strategies may lead to the hypothesis that both weight loss and the psychosomatic component of the disease could have a major role in the disease. In addition, all of these diets are generally regarded as healthy dietary models, rich in plant foods, antioxidants or fiber, so the fact that people have experienced an improvement in symptoms after almost all dietary interventions suggests that an adequate diet could play a crucial role in the management of FM. However, these results should be interpreted with caution since the aforementioned studies present several biases that limit the robustness of the findings. First of all, most studies have a limited sample size with no possibility of blinding due to the nature of dietary intervention trials. Secondly, outcomes are often analyzed using different methodologies and without considering possible confounding factors. In addition, adherence to the assigned dietary intervention is hardly ever evaluated. Finally, a follow-up is almost never carried out to determine whether the positive effects are maintained over time or are only transient. Therefore, although dietary aspects appear to be a promising complementary approach to treat FM, further research is needed to improve the understanding of the disease and to provide the most effective strategies for managing FM syndrome.

## Figures and Tables

**Table 1:** Nutritional supplementation in fibromyalgia (FM) subjects.

Author, Year	Country	Intervention, n	Control, n	Age, y	Sex	Duration	Intervention	Control	Outcomes	Findings	Efficacy *
**Vitamin D**											
Warner et al., 2008 [24]	US	25	25	58.0 ± 7.3 intervention; 56.7 ± 11.3 control	F	12 weeks	50,000 IU of oral ergocalciferol weekly	Placebo	25(OH)D, duration of pain, VAS, FPS	25(OH)D increase in the intervention group; no difference in duration of pain, VAS and FPS score	=
Arvold et al., 2009 [25]	US	48	42	59.7 ± 14.0 intervention; 57.8 ± 15.8 control	All	8 weeks	50,000 IU of oral cholecalciferol weekly	Placebo	25(OH)D, PTH, creatinine, calcium, self-reported symptoms, FIQ	25(OH)D increase and PTH decrease in the intervention group. 5 out 20 FIQ items and total FIQ score improved after intervention. Severely deficient patients did not show symptom improvement	+
Abokrysha et al., 2012 [26]	Saudi Arabia	30	NA	34.6 ± 8.1	F	8 weeks	600,000 IU of intramuscular single dose or 50,000 IU oral cholecalciferol weekly	NA	WPI, fatigue, waking unrefreshed, cognition, SS	Improvement of WPI, fatigue, waking unrefreshed and SS score after treatment	+
Wepner et al., 2014 [27]	Austria	15	15	48.4 ± 5.3	All	20 weeks	2400 IU or 1200 IU (according to serum calcifediol levels) of cholecalciferol daily	Placebo	Calcifediol, pain severity (VAS), SF-36; HADS-D, FIQ, SCL-90-R	Severity of pain and physical role functioning scale improved after intervention	+/=
Yilmaz et al., 2016 [28]	Turkey	30	NA	36.9 ± 9.2	All	12 weeks	50,000 IU of oral cholecalciferol weekly	NA	Ca, P, ALP, 25(OH) D, pain severity (VAS), asthenia (VAS), TPC, BDI, SF-36, waking unrefreshed, headache, tenderness on tibia	Marked decrease in pain, asthenia, severity of waking unrefreshed, TPC, and BDI and improvement in quality of life after treatment	+
Dogru et al., 2017 [29]	Turkey	42	28	38.7 ± 5.2	F	12 weeks	50,000 IU of oral cholecalciferol weekly	No treatment	FIQ, SF-36, pain severity (VAS), ASEX, BDI	Improvements in physical function, physical role limitations, emotional role limitations, social function, mental health, vitality, and quality of life after treatment	+
de Carvalho et al., 2018 [30]	Brazil	11	NA	48.5 (28-67)	F	12 weeks	50,000 IU of oral cholecalciferol weekly	NA	25(OH)D, pain severity (VAS), TPC	Improvements in 25(OH)D levels, pain severity and reduction in TPC	+
Mirzaei et al., 2018 [31]	Iran	37	37	42.1 ± 10.8 intervention; 41.0 ± 10.3 control	All	8 weeks	Trazodone 25 mg + 50,000 IU of oral cholecalciferol weekly	Trazodone 25 mg + placebo	25(OH)D, WPI, FIQ, PSQI, SF-36	Improvement in 25(OH)D, WPI, FIQ, PSQI and SF-36 in the intervention group	+
**Vitamin C + E**											
Naziroglu et al., 2010 [32]	Turkey	21 (*n* = 11 vit. C + E; *n* = 10) vit. C+E + exercise)	11	40.5 ± 4.9 vit. C + E; 37.4 ± 4.0 vit. C + E + exercise; 37.8 ± 8.7 control	F	12 weeks	150 mg/day of α -tocopheryl acetate and 500 mg/day ascorbic acid or 150 mg/day of α -tocopheryl acetate and 500 mg/day ascorbic acid + exercise	Exercise	Vitamin A, C and E, β-carotene, LP, GSH, GSH-Px, pain severity (VAS)	Improvement of LP, GSH, GSH- Px and plasma vitamins A, C, and E after the supplementations with or without exercise	+/=
**Magnesium**											
Russell et al., 1995 [33]	US	12	12	49	F	4 weeks	200 mg malic acid + 50 mg magnesium, 3 tablets/day up to 6 tablets/day	Placebo	Pain severity (VAS), TPC, TPA, HAQ, CESD, Hassle, psychological response to events	Little or no effect with low doses; improvements in the severity of primary pain/tenderness measures with dose escalation and a longer duration of treatment	+/=
Bagis et al., 2013 [34]	Turkey	40 (*n* = 20 Mg citrate; *n* = 20 Mg citrate + amitriptyline)	20	40.2 ± 5.1 Mg citrate;40.7 ± 5.2 Mg citrate + amitriptyline; 42.1 ± 6.2 control	F	8 weeks	300 mg/day of Mg citrate or 300 mg/day of Mg citrate + 10 mg/day amitriptyline	10 mg/day amitriptyline	Pain severity (VAS), TPC, FIQ, BDI, BAI, self-reported symptoms	Improvement in TPC, FIQ and BDI with the Mg citrate treatment. Improvement in almost all parameters except for pain, FIQ, headache, gastric problems, IBS, cramps after amitriptyline treatment. Improvement in all parameters except numbness after the combined amitriptyline + Mg citrate treatment	+
**Iron**											
Boomershine et al., 2018 [35]	US	41	40	41.2 ± 11.1 intervention; 43.9 ± 10.8 control	All	6 weeks	15 mg/kg (up to 750 mg) of ferric carboxymaltose for 5 days	Placebo	Iron indices, hematology parameters, FIQR, BPI, MOS Sleep scale, Fatigue VNS	Improvement in FIQ, BPI, fatigue and iron indices in the treatment group.	+
**Probiotics**											
Roman et al., 2018 [36]	Spain	20	20	55.0 ± 8.4 intervention; 50.3 ± 7.9 control	All	7 weeks	4 pills/day containing *Lactobacillus Rhamnosus GG^®^, Casei, Acidophilus, and Bifidobacterium Bifidus*	Placebo	Pain severity (VAS), FIQ, SF-36, BDI, STAI, MMSE, cortisol	Improved impulsivity and decision-making after the intervention	+/=

ALP—alkaline phosphatase; ASEX—Arizona sexual life questionnaire; BAI—Beck Anxiety Inventory; BDI—Beck Depression Inventory; BPI—Brief Pain Inventory; CESD—Center for Epidemiologic Studies Depression Scale score; FIQ—Fibromyalgia Impact Questionnaire; FIQR—Revised Fibromyalgia Impact Questionnaire; FPS—Functional Pain Score; GSH—glutathione; GSH-Px—glutathione peroxidase; HADS-D—Hospital Anxiety Depression Scale Deutsche version; HAQ—Health Assessment Questionnaire; Hassle—Hassle Scale score; LP—lipid peroxidation; MMSE—Mini Mental State Examination; MOS—Medical Outcomes Study Sleep Scale; NA—Not applicable; PSQI—Pittsburgh Sleep Quality Index; PTH—parathyroid hormone; SCL-90-R—Symptom Checklist-90-Revised; SF-36—Short Form Health Survey; SS—Symptom severity score; STAI—State Trait Anxiety Inventory; TPA—tender point average; TPC—tender point count; VAS—Visual Analogue Scale; VNS—Visual Numeric Scale; WPI—Widespread Pain Index. * + significant improvement in (almost) all the investigated outcomes; = no significant improvement in the investigated outcomes.

**Table 2:** Dietary interventions in FM subjects.

Author, Year	Country	Intervention, n	Control, n	Age, y	Sex	Duration	Intervention	Control	Outcomes	Findings	Efficacy *
**Olive oil**											
Rus et al., 2017 [51]	Spain	11	12	53.6 ± 5.5 intervention; 48.2 ± 8.0 control	F	3 weeks	50 mL/die EVOO	50 mL/die ROO	BMI, SBP, DBP, cardiac frequency, oxidative stress markers, antioxidative markers, FIQ, pain severity (VAS), PCS-12, MCS-12	Improvement in protein carbonyls, lipid peroxidation, FIQ and mental health status after the intervention with EVOO	+
Rus et al., 2020 [52]	Spain	15	15	54.1 ± 5.6 intervention; 49.8 ± 5.8 control	F	3 weeks	50 mL/die EVOO	50 mL/die ROO	weight, BMI, waist circumference, thrombosis-related parameters, ESR, inflammatory markers, NO, lipid profile, cortisol	EVOO declined red blood cell count and ESR. ROO increased mean platelet volume and reduced PDW, neutrophil-to-lymphocyte ratio, ESR and fibrinogen. Significant differences in pre–post change between EVOO and ROO for cortisol and PDW	+
**Ancient grain**											
Pagliai et al., 2020 [53]	Italy	10	10	46.2 ± 11.5 intervention; 51.7 ± 12.9 control	All	8 weeks	Pasta, bread, cracker, biscuits made with ancient grain Khorasan	Pasta, bread, cracker, biscuits made with modern grain Palesio	WPI, SS, FIQ, FSS, TSS, SRSBQ, RSQD, FOSQ	Improvement in WPI + SS, FIQ and FOSQ after the intervention	+
**MSG and aspartame-free diet**											
Holton et al., 2012 [54]	US	46	NA	53.0 ± 13.0	All	4 weeks	MSG and aspartame-free diet	NA	28-symptom checklist, pain severity (VAS), FIQR, IBS QOL	Improvement in all the tested outcomes after the intervention	+
Vellisca et al., 2014 [55]	Spain	36	36	42.3 ± 8.4 intervention; 39.6 ± 8.2 control	F	12 weeks	MSG and aspartame-free diet	Free diet	Pain severity (VAS)	No significant differences in pain referred after the intervention	=
**Gluten-free diet**											
Rodrigo et al., 2013 [56]	Spain	7	NA	49.0 ± 12.0	F	1 year	Gluten-free diet	NA	TPC, FIQ, HAQ, SF-36, gastrointestinal complaints (VAS), pain severity (VAS), fatigue (VAS), tTG	Improvement of all the tested outcomes after the intervention	+
Rodrigo et al., 2014 [57]	Spain	97	NA	50.0 ± 8.0	All	1 year	Gluten-free diet	NA	TPC, FIQ, HAQ, SF-36, gastrointestinal complaints (VAS), pain severity (VAS), fatigue (VAS)	Improvement of all the tested outcomes after the intervention only in the lymphocytic enteritis subgroup	+
Isasi et al., 2014 [58]	Spain	20	NA	46 (25–73)	F	16 months	Gluten-free diet	NA	Widespread pain, return to work, return to normal life	Improvement of all the tested outcomes after the intervention	+
Slim et al., 2017 [59]	Spain	35	40	52 (36–66) intervention; 53 (32–65) control	F	24 weeks	Gluten-free diet	Hypocaloric diet	NCGS symptoms, BMI, waist circumference, FIQR, PSQI, BPI-SF, BDI, STAI, SF-12, PGI-S	No statistically significant difference in the tested outcomes between intervention and control treatment	=
**Low-FODMAPs diet**											
Marum et al., 2016 [60]	Portugal	38	NA	38.5 ± 10.0	F	4 weeks	Low-FODMAPs diet	NA	FSQ, FIQR, IBS-SSS, EQ-5D, abdominal and somatic pain (VAS), satisfaction	Improvements in VAS, FSQ, FIQR and GI symptom	+
Marum et al., 2017 [61]	Portugal	38	NA	38.5 ± 10.0	F	4 weeks	Low-FODMAPs diet	NA	Body weight, BMI, body composition, waist circumference	Weight, BMI and waist circumference decreased after the intervention, but no significant effect on body composition	+
**Low-calorie diet**											
Shapiro et al., 2005 [62]	US	42	NA	54.5 ± 8.1	F	5 months	Hypocaloric diet (1200–1500 kcal/die)	NA	Body weight, BMI, waist circumference, FIQ, HAQ, MPI, BDI-II, STAI, QOL, BSQ	Improvement in pain, body image, anxiety, quality of life and depression after the intervention	+
Senna et al., 2012 [63]	Egypt	43	43	44.8 ± 13.6 intervention; 46.3 ± 14.4 control	All	6 months	Hypocaloric diet (1200 kcal/die: 50% CHO, 30% Fat, 20% Protein)	Isocaloric diet	FIQ, TPC, BDI-II, PSQI, Body weight, BMI, waist circumference, IL-6, CRP	Improvements in pain, fatigue, depression, IL-6, CRP	+
Schrepf et al., 2017 [64]	US	123	NA	50.7 (23–69)	All	12 weeks	Hypocaloric diet (800 kcal/die)	NA	Body weight, WPI, SS IDS, inflammatory markers	Improvements in pain, symptom severity, depression, FM scores, IL-10 after weight loss	+
**Vegetarian diet**											
Hostmark et al., 1993 [65]	Norway	10	NA	49.9 ± 4.1	All	3 weeks	Vegetarian diet	NA	Peroxides, lipid profile, apolipoproteins, fibrinogen	Improvements in serum peroxide concentration, fibrinogen, total cholesterol, apolipoprotein-B and -A	+
Azad et al., 2000 [66]	Bangladesh	37	41	NA	All	6 weeks	Vegetarian diet	Amitriptyline	Fatigue, insomnia, non-restorative sleep, pain severity (VAS), TPC	No statistically significant difference in the tested outcomes between intervention and control treatment	=
Kaartinen et al., 2000 [67]	Finland	18	15	51 (34–62) intervention; 52 (37–59) control	F	12 weeks	Raw vegan diet	Omnivorous diet	BMI, HAQ, TPC, pain severity (VAS), BDI, sleep, haematocrit, ESR, total cholesterol, urinary Na, GHQ, physical activity	Improvements in pain, autonomy, sleep quality, morning stiffness, total cholesterol and urinary Na after the intervention	+
Hänninen et al., 2000 [68]	Finland	33	20	NA		12 weeks	Raw vegan diet	Omnivorous diet	Antioxidants, lignan, carotenoids, vitamins, morning stiffness, pain severity (VAS)	Improvements of carotenoids, phenolic compounds, vitamin C and E, joint stiffness, pain, general well-being after the intervention	+
Donaldson et al., 2001 [69]	US	30	NA	NA	All	7 months	Raw vegan diet	NA	FIQ, SF-36, QOL, physical performance	Improvement in pain, vitality, mobility, general well-being after the intervention	+
Martínez-Rodríguez et al., 2018 [70]	Spain	14 (*n* = 7 LOV; *n* = 7 LOV + exercise)	7	34.0 ± 2.0 LOV + exercise; 34.0 ± 2.0 LOV; 33.0 ± 3.0 control	F	4 weeks	LOV or LOV + exercise	Free diet and no exercise	Pain severity (VAS), body composition	Improvement in body composition and pain severity after the intervention with diet and exercise	+
**Mediterranean diet**											
Michalsen et al., 2005 [71]	Germany	14	21	51.6 ± 13.3 intervention; 52.0 ± 10.0 control	All	8 weeks	Mediterranean diet	8-days fasting	Gut microbiota composition, stool pH, IgA, pain severity (VAS)	No statistically significant difference in the tested outcomes between intervention and control treatment	=
Martínez-Rodríguez et al., 2020 [72]	Spain	11	11	48.0 ± 4.0 intervention; 50.0 ± 5.0 control	F	16 weeks	Mediterranean diet + 60 mg of tryptophan and 60 mg of Mg	Mediterranean diet	PSQI, BSQ, STAI, POMS-29, EAT-26	Improvements in anxiety, mood disturbance, eating disorders, dissatisfaction with body image after tryptophan and Mg-enriched Mediterranean diet	+

BDI—Beck Depression Inventory; BPI-SF—Brief Pain Inventory-short form; BMI—Body Mass Index; BSQ—Body Shape Questionnaire; CRP—C-reactive protein; DBP—Diastolic blood pressure; EAT-26—Eating Attitude Test-26; EQ-5D—Euro-QOL quality of life instrument; ESR—erythrocyte sedimentation rate; EVOO—Extra-virgin olive oil; FIQ—Fibromyalgia Impact Questionnaire; FSQ—Fibromyalgia Survey Questionnaire; FSS—Fatigue Severity Scale; GHQ—General Health Questionnaire; HAQ—Stanford Health Assessment Questionnaire; IBS QOL—Inflammatory Bowel Disease Quality of Life; IBS-SSS—Irritable Bowel Syndrome-Symptom Severity Survey; IDS—Inventory of Depressive Symptomology; IL—Interleukin; LOV—Lacto-ovo-vegetarian diet MCS-12—Mental Component Summary of the Short Form-12 Health Survey; MPI—West Haven-Yale Multidimensional Pain Inventory; MSG—Monosodium-glutamate; PSC-12—Physical Component Summary of the Short Form-12 Health Survey; NA—Not applicable; NCGS—Non-celiac gluten sensitivity; PDW—platelet distribution width; PGI-S—Patient Global Impression scales of severity; POMS-29—Profile of Mood States Questionnaire; PSQI—Pittsburgh Sleep Quality index; QOL—Quality Of Life Survey; ROO—Refined olive oil; RSQD—Restorative Sleep Questionnaire–Daily; SBP—Systolic blood pressure; SF-12—Short Form Health Survey; SF-36—Short Form Health Survey; SRSBQ—Sleep-Related and Safety Behaviour Questionnaire; SS—Symptom Severity scale; STAI: State Trait Anxiety Inventory; TPC—tender point count; TSS—Tiredness Symptoms Scale; tTG—tissue-Trans-Glutaminase; VAS—Visual analogue scale; WPI—Widespread Pain Index. * + significant improvement in (almost) all the investigated outcomes; = no significant improvement in the investigated outcomes.
